# Mesenchymal Stem Cell Secretome as an Emerging Cell-Free Alternative for Improving Wound Repair

**DOI:** 10.3390/ijms21197038

**Published:** 2020-09-24

**Authors:** Parinaz Ahangar, Stuart J. Mills, Allison J. Cowin

**Affiliations:** 1 Future Industries Institute, University of South Australia, Adelaide, SA 5000, Australia; parinaz.ahangar@mymail.unisa.edu.au (P.A.); stuart.mills@unisa.edu.au (S.J.M.); 2 Clinical and Health Sciences, University of South Australia, Adelaide, SA 5000, Australia

**Keywords:** mesenchymal stem cells, secretome, wound healing

## Abstract

The use of mesenchymal stem cells (MSC) for the treatment of cutaneous wounds is currently of enormous interest. However, the broad translation of cell therapies into clinical use is hampered by their efficacy, safety, manufacturing and cost. MSCs release a broad repertoire of trophic factors and immunomodulatory cytokines, referred to as the MSC secretome, that has considerable potential for the treatment of cutaneous wounds as a cell-free therapy. In this review, we outline the current status of MSCs as a treatment for cutaneous wounds and introduce the potential of the MSC secretome as a cell-free alternative for wound repair. We discuss the challenges and provide insights and perspectives for the future development of the MSC secretome as well as identify its potential clinical translation into a therapeutic treatment.

## 1. Introduction

Despite advances in our understanding of the mechanisms involved in acute and chronic wound repair, non-healing wounds remain a cause of morbidity and mortality worldwide and are a huge economic burden to our society [1]. Generally, cutaneous wounds heal through an intricate cascade of phases in which the interactions of different cell types alongside local and systemic factors replace injured tissues and re-establish supportive structures [2]. However, when these processes fail to progress normally and in conjunction with an underlying disease state, chronic non-healing wounds may eventuate [3]. The best practice in wound management is aimed at promoting healing and preventing complications, such as scarring. However, despite the plethora of wound products available on the market, there remains a significant number of wounds that either fail to heal or heal with scarring. There is, therefore, a clear need for the development of alternative wound therapies that promote healing and reduce scar formation.

It has been suggested that cell-based therapies have great potential for the treatment of wounds. Stem cells have been shown to accelerate the healing process, and it has been proposed that these cells can induce regenerative healing rather than the repair mechanisms that result in scar formation [4]. Direct incorporation into regenerating tissues and differentiation to parenchymal cells has been hypothesised to be the main mechanism by which mesenchymal stem cells (MSC) exert their beneficial effects [5,6]. However, it has been shown more recently that the rate of MSC survival, engraftment and the number of newly generated cells, by cell fusion or differentiation, seems to be too low to explain the significant effects achieved by MSCs [7,8]. Proteomic analysis of MSC conditioned media (MSC-CM), containing MSC secretome (MSC-S), shows that stem cells secrete a broad range of biologically active molecules, including cytokines, mRNAs, growth factors and active lipids with vital roles in skin tissue regeneration [9]. Hence, paracrine signalling of MSCs has been suggested as the main mechanism of action [10]. This breakthrough in the field of MSCs has motivated researchers to investigate the application of the MSC-S on wound healing to overcome the challenges of using live cells. This review describes the potential effects of MSC-S on cutaneous wound healing and additionally discusses the challenges in translating its use into a therapeutic treatment.

## 2. MSCs as A Cell Therapy for Cutaneous Wound Healing

MSCs are non-hematopoietic and plastic-adherent cells that exhibit a fibroblast-like phenotype [11]. They are a heterogeneous population that was first discovered in the bone marrow (BM-MSCs) [12], but later, they were obtained from various adult tissues, such as adipose tissues (ADSCs) [13], placenta (p-SCs) [14], dental pulp (DPSCs) [15] and umbilical cord (UC-MSCs) [16]. MSCs are able to renew themselves and differentiate into various tissue-forming cell lineages, such as chondrocytes, adipocytes, osteocytes, liver epithelium, endothelial cells, smooth muscle cells and keratinocytes [17,18,19]. MSCs stain positive for cluster of differentiation 44 (CD44), CD90, CD105 and CD73, and negative for CD11b or CD14, CD19 or CD79α, CD34, CD45 and HLA-DR [20,21]. MSCs are considered as immune-privileged cells since they do not express the major histocompatibility complex (MHC) II and costimulatory molecules, such as CD86, CD40 or CD80 and express a low level of MHC I [22]. MSCs also possess immunomodulatory properties that can alter the function of T cells, B cells, natural killer (NK) cells and monocytes/macrophages [23]. Overall, these properties suggest that MSCs could potentially revolutionise cell therapies for the regeneration of damaged tissue in many different systems, such as cardiac, bone, kidney and lung [24,25]. 

Treatment of wounds with MSCs has been shown to have beneficial effects, including the acceleration of wound closure [26]. MSCs differentiate into various tissue-specific cell types, which can promote angiogenesis, suppress the immune system, and secrete and remodel the extracellular matrix (ECM) [8,25]. MSCs exhibit reparative, regenerative and immunomodulatory effects through paracrine signalling, pointing towards the promising therapeutic potential of these cells [27]. Indeed, numerous studies have shown that the administration of MSCs to cutaneous wounds enhances the healing of skin injuries, including acute and diabetic wounds and burns in mice, rats and pigs. MSCs that are derived from different tissues possess differences, which are mainly reflected in the expression of marker genes, proliferation rate, differentiation capacity, secreted cytokine profile and immunomodulatory capacity [28,29]. Treatment with MSCs supports wound healing by accelerating re-epithelialisation, improving granulation tissue formation, stimulating angiogenesis and diminishing inflammation [30]. These consistent and promising results have led to the use of MSCs in clinical trials as human wound therapies. In these trials, autologous BM-MSCs have been administered to chronic cutaneous ulcerations [31], diabetic foot ulcers [32], presser ulcers [33], radiation burns [34], resulting in accelerated wound closure and improved healing properties. All these findings from preclinical and clinical studies demonstrate that MSCs could be a promising resource for regenerative therapy [35].

## 3. Development of MSC Secretome as An Alternative Cell-Free Therapy for Cutaneous Wounds

Recent studies have suggested that the main therapeutic benefits of MSCs are not limited solely to their cell-to-cell interactions [36,37,38,39]. MSCs secrete a broad range of bioactive molecules, including proteins, nucleic acids, proteasomes, exosomes, microRNA and membrane vesicles, collectively known as the secretome, in response to the surrounding environment [40,41]. The MSC secretome (MSC-S) then influences neighbouring cells and regulates multiple biological processes [42]. Currently, paracrine or trophic properties are considered as the primary means of the therapeutic effect of MSCs [26,40,43]. Although MSCs derived from different organs share phenotypic and regenerative characteristics, their secretome is different and depends on their origin, which consequently can lead to different therapeutic potentials [44]. MSC-S from various origins has been used to assess its effect on skin cell functionality as well as its effects on wound healing using in vitro and in vivo models (summarised in Table 1). 

## 4. Potential Mechanism of Action of MSC-S

**Figure 1:**
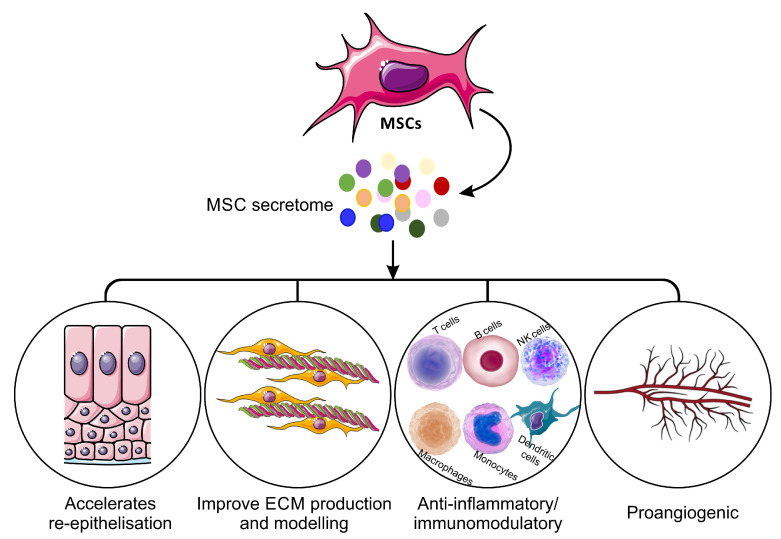
Mechanisms of mesenchymal stem cells secretome on the wound healing process.

The mechanism of action of the MSC-S must be elucidated before it can be widely introduced as a potential new therapy in the clinic. Recent advances in cell and molecular biology have offered insights into multiple mechanisms, and it has been proposed that MSC-S can contribute to wound healing (Figure 1). Dissection of the MSC-S shows a large repertoire of proteins known to be involved in skin inflammation, haemostasis and wound repair (Table 2). The biochemical pathways and mechanism of action of these proteins have been shown previously [36,40,61,62]. 

The MSC-S is, therefore, a complex mixture of bioactive factors and has been shown to have significant positive effects in the treatment of inflammatory disorders of nervous, cardiovascular, respiratory and skeletal systems [63,64,65]. Although it is believed that the anti-inflammatory effects of cells rely on direct cell–cell interactions, several studies have demonstrated that the interaction between MSCs and immune cells can be attributed to MSC-secreted cytokines [66]. For example, MSC-secreted interleukin-1 receptor antagonist (IL1-RA) inhibits B cell differentiation [67]. Human MSC-derived galectin-1 also has inhibitory impacts on the proliferation of alloreactive CD4+ and CD8+ T cells [68]. MSCs also secrete programmed death-ligand 1 (PD-L1), which suppresses T cell activation and increases T cell apoptosis [69,70]. Furthermore, MSC-secreted Prostaglandin E2 (PGE2), TGF-β1, IL-6 and nitric oxide, all provide inhibitory effects on T cells, macrophages, neutrophils and NK cells [71,72]. MSC-S as a whole has been shown to exert immunosuppressive effects through modulating proliferation and activation of immune cells in vitro [51]. Treatment of peripheral blood mononuclear cells with MSC-S led to a reduction in pro-inflammatory cytokine production and an increase in anti-inflammatory cytokines [54]. BM-MSC-S injected into the margins of excisional wounds in mice promotes wound healing through diminished inflammation mediated by macrophage polymerisation [36]. This beneficial effect of MSC-S is significantly higher than the equivalent treatment with fibroblast secretome [36].

Increased angiogenesis has been proposed as another of the main mechanisms of action for MSC-S in different types of wounds supported by in vitro treatment of endothelial cells with MSC-S enhancing their proliferation and migration. [37,38]. This impact of MSC-S on angiogenesis is suggested to be mediated by the secretion of Cyr61 from MSCs [50]. Pro-angiogenic proteins secreted by MSCs, such as Ang-1, Ang-2, VEGF, angiostatin, CXCL16, EGF, FGF, PDGF, granulocyte-macrophage colony-stimulating factor (GM-CSF), HGF, MCP-1, MMP-8 and MMP-9, also contribute to vascular formation and stability [73]. In preclinical studies, BM-MSC-S treatment improved partial-thickness burn injury repair in rats, which was mediated by increased blood vessel formation [37]. In another study, topical administration of BM-MSC-S cream to full-thickness burns of rats resulted in increased numbers of fibroblasts and improved angiogenesis as well as accelerated wound closure [38]. Subcutaneous injection of umbilical cord-derived MSC secretome (UC-MSC-S) to wounds of diabetic mice led to accelerated wound closure and high capillarity density in wound areas [57].

MSC-S from different origins (such as iliac crest, bone marrow, adipose, Wharton’s jelly, umbilical cords) have been shown to enhance the migratory and proliferative abilities of dermal fibroblasts and epidermal keratinocytes in vitro [45,46,47,74]; MSC-S alters the expression of genes involved in re-epithelialisation and angiogenesis and increases re-epithelialisation in human 3D skin models [48,49]. The secretome from adipose tissue-derived MSCs (ADSC-S) has been shown to protect dermal fibroblasts from oxidative stress-mediated apoptosis and accelerate wound closure with stimulatory effects on fibroblast migration in in vitro models [52,53]. The beneficial effect of MSC-S on skin cells is believed to be mediated by growth factors (such as IGF-1, EGF, FGF-2, KGF, TGF-β, HGF, PDGF, VEGF, SDF-1, erythropoietin) and chemokines (such as IL-6, IL-8, MCP-1 and RANTES) (Table 1) [36,40,61,62,74,75,76]. Treatment of wounds with MSC-S significantly accelerates new tissue formation, collagen deposition and re-epithelialisation in treated wounds [36,49,55,56]. Application to chronic rat wounds of BM-MSC-S delivered in a fibrin vehicle also increases re-epithelialisation and collagen deposition [39]. In another study, excisional wounds of rats topically treated with ADSC secretome displayed accelerated wound closure along with faster re-epithelialisation [55]. It has further been demonstrated that dental pulp stem cell (DPSC) secretome enhances wound healing through increased collagen synthesis and improved proliferative and migratory ability of dermal fibroblasts [56]. MSC-S derived from Wharton’s jelly (WJ-MSC-S) promotes excisional wound healing in mice through increased cell proliferation [58]. Recently, WJ-MSC-S has also been shown to promote wound healing in radiation-induced cutaneous wounds of rats [59].

## 5. Advantages of MSC-S over Other Cell-Based Products 

The use of cell-based therapies and products is not new. Indeed, skin substitutes, platelet-rich plasmas, recombinant growth factors and cytokines have been around for decades [1]. However, despite promising preclinical datasets and successful clinical trials, there remains the need for improved cell-based solutions, as evidenced by the spiraling increase in chronic wounds worldwide. Currently, skin substitutes containing living fibroblasts, keratinocytes or both, include TransCyte, [77], Dermagraft [78], Apligraf, [79] and OrCel [80]. These cell-based skin graft substitutes have shown promising results in promoting faster wound closure (Transcyte), improved rate of re-epithelialisation (Dermagraft), and superior vascularity, pigmentation, wound height and scar scores (Apligraf) [81]. However, they are expensive, require specific storage conditions, have the potential risks of tumorigenicity, infection and rejection, and are difficult to use within the community [82]. Recombinant growth factors were postulated to be the solution to impaired healing as it was hypothesised that chronic non-healing wounds lacked specific growth factors and cytokines [83]. Numerous clinical trials were undertaken to investigate the growth factor therapies including EGF, PDGF, GM-CSF, KGF therapies, but despite appearing to be efficacious in many animal models of wound repair, translation into clinical products has been limited due to the significant amounts of growth factors required for treatment, the expense of manufacture and the lack of clinically relevant improvements in healing [84,85,86,87]. To date, only PDGF has received FDA approval for the treatment of diabetic foot ulcers, and its use is limited due to the need for dressing replacement and the potential increased risk of malignancy [88,89]. Administering single growth factors and/or cytokines has potential limitations as wounds are complex environments, and multiple factors may be required to stimulate healing responses. MSC-S contains a vast array of proteins at physiological and balanced levels, including cytokines, growth factors and chemokines (Table 2), that potentially makes it a superior alternative to expensive cytokine and growth factor therapies that are limited to delivering only one or two proteins to the wounds. 

The delivery of live cells to cutaneous wounds presents a unique and specific set of challenges [90]. The injection of cells through a syringe or needle has been shown to decrease cell viability to only 1–32% and can cause irreversible and sometimes fatal damage to the cell membrane [91,92]. Not only does this negate the potential benefit of the cell therapy but the introduction of a large population of apoptotic or necrotic cells may serve to elicit an immune response, which could be detrimental to the healing process. MSC-S therapy avoids the difficulties associated with live-cell administration in stem cells as well as advantages of ease of mass production, packaging and transportation [62]. These advantageous factors have led to the growing potential of MSC-S use as a treatment for tissue regeneration and various disorders [40]. 

## 6. Challenges with the MSC-S as a Wound Therapy

### 6.1. Secretome Characterisation

Although MSC-S may be a promising medical product, it has been very challenging to define its biochemical composition or measure the activity and half-life of all of its components [62]. In addition to proteins, MSC-S also contains exosomes and extracellular vesicles [93]. Exosomes can contain miRNA, lipids and long noncoding RNAs, which regulate multiple signalling pathways related to inflammation [94]. The identification and characterisation of all the biomolecules that constitute the secretome are difficult to achieve but will improve the understanding of the secreted factor profile and provide information about its function, regulation and clinical use [95]. Further research on the MSC-S using high throughput genetic and chemical screenings and next-generation metabolomics-driven approaches is required to clarify all of the key metabolic and signalling pathways that are mediating robust new tissue formation, dampened inflammation and enhanced wound closure.

### 6.2. Inconsistency in Preparation of Secretome

It has been reported that the isolation and culture methods, as well as donor health condition and age, can affect the quality of MSC products [96]. Inconsistency in secretome harvesting in terms of MSC heterogenicity, inter-donor differences, cell number and time interval is another part of the current challenge regarding the clinical use of secretome. Production of MSC-S under pharmaceutical standards and according to good manufacturing practice (GMP) is a vital step to use MSC-S as a therapeutic agent in the clinic. Compliance with well-defined good manufacturing protocols (GMP) will improve batch-to-batch consistency and the reproducible efficacy of MSC-S [97]. 

### 6.3. Potential Side Effects of MSC-S

Although there are limited reports of the negative effects of secretome, there are always potential risks using exogenous biological molecules, although these risks are reduced when compared to cell-based therapies. A comprehensive analysis is needed before MSC-S transplantation to specific niches in different tissues. For example, MSC-S contains MSC-derived exosomes and extracellular vesicles that can be immunogenic; however, the immunogenicity of exosomes has been shown to be less than their parent MSCs [98]. On the other hand, immunosuppressive properties of MSC-S have been reported in several studies and have been hypothesised to be one of the main mechanisms of action of MSC-S when treating autoimmune diseases [99]. However, the use of secretome may diminish the immune system, which may increase the risk of infection, immunodeficiency and tumour growth in treated patients [100]. Thus, an optimal amount of secretome should be clearly defined with an aim to find the right balance between safety and effectiveness of any secretome based therapy.

### 6.4. Limitation of Secretome Resources and Instability of Secretome Components

The number of MSCs that are required to produce sufficient quantities of secretome for an equivalent effect on acute wounds is about 10–25 times higher than directly administered live cells [43]. These elevated numbers of cells impact the costs of derivation and validation because the biological properties and activity of these cells may change with repeated passages. However, with increased production and improvement in cell factories and bioreactors, the impact of this drawback may be minimised. Another major concern in secretome therapy is the instability and short half-life of proteins. One of the successful strategies to address these drawbacks is preconditioning cells to stimulate the paracrine production of the secretome. Preconditioning is also useful to control the composition of the secretome to avoid the toxicity caused by upregulated cytokines [95]. Hence, it is important to first elevate the production of desirable factors and downregulate the detrimental ones, and second to achieve an appropriate balance between stimulatory and inhibitory factors produced by these cells. There are different pre-treatment methods for MSCs, for example, subjecting cells to hypoxia or anoxia has been reported to increase the secretion of cytokines and growth factors in transplanted stem cells [101]. Genetic manipulation of cells using transgenes can also alter specific gene expression with the aim of controlling the MSC-S post-transplantation [102]. Another promising approach for pre-treating stem cells before transplantation involves small molecules, such as inflammatory cytokines and growth factors [103]. For example, treating MSCs with inflammatory cytokines increases their secretion of anti-inflammatory biomolecules and improves their immunosuppressive function [104]. Preconditioning through cell–cell interactions is another strategy to improve the secretion of favourable biomolecules. For example, Potapova et al. (2007) reported that MSCs in 3D spheroids are able to secrete higher levels of paracrine biomolecules, such as IL-11, VEGF, FGF-2 and angiogenin, compared to MSCs in monolayers [105]. This tailoring of the MSC-S could potentially lead to numerous off the shelf products specifically designed for the treatment of specific conditions or wound types.

## 7. Conclusions

Even though advances in the field of stem cell therapy have grown significantly, there are still practical and clinical hurdles to overcome before they can be routinely used for the treatment of wounds. Poor engraftment and survival of cells in damaged areas, immunogenicity, tumorigenicity and lack of efficiency are notable limitations for clinical stem cell therapies. The use of MSC-S as a potential alternative to MSCs is of enormous interest and has significant clinical potential, given the trophic properties of many of the secreted factors. MSC-S therapy avoids the use of live cells and can limit biological variability, therefore, leading to the potential development of efficient and safe therapeutic approaches. While the mechanism of action of the MSC-S is still to be fully determined, the development of a cell-free therapy for the treatment of cutaneous wounds holds great promise. 

## Figures and Tables

**Table 1:** Therapeutic potential of the MSC secretome (MSC-S) in cutaneous wound healing.

MSC-S Origin	Target Cell/Wound Type	Outcome	Effective MSC-S Component	Reference
Human iliac crest MSC	Fibroblasts and keratinocytes	Accelerated migration of skin cells.	Transforming growth factor β1 (TGF-β1), Monocyte Chemoattractant Protein-1 (MCP-1), Interleukin-6 (IL-6), IL-8, collagen I, fibronectin and insulin-like growth factor-binding protein (IGFBP)	[45]
Human UC-MSC	Fibroblasts	Increased proliferation and migration. Increased expression of genes involved in scar-less healing. Secrete less TGF-β and more matrix metalloproteinase (MMP)/ Tissue inhibitor of metalloproteinase TIMP.	Not identified	[46]
Human ADSC	Fibroblasts	Stimulated collagen secretion and ECM production. Upregulated migration.	Not identified	[47]
Human hip joints MSC	Humanised 3D skin model	Increased migration of the epidermal layer.	Keratinocyte growth factor (KGF), hepatocyte growth factor (HGF), platelet derived growth factor (PDGF), stromal cell-derived factor-1 (SDF-1)	[48]
Mouse BM-MSC	Fibroblasts	Increased proliferation and accelerated migration. Downregulated Intercellular adhesion molecule 1 (ICAM1), Vascular cell adhesion protein (VCAM1) and MMP11.	Not identified	[49]
Human embryonic stem cells derived MSC	Endothelial cells	Increased angiogenesis. Induced morphogenesis of endothelial cells.	Cysteine-rich angiogenic inducer 61(Cyr61)	[50]
Mouse BM-MSC	CD^+^ T cells	Decreased T cell proliferation.	Not identified	[51]
Human ADSC	Fibroblasts	Antiapoptotic effect and antioxidant effect.	Superoxide dismutase (SOD), Insulin-like growth factor (IGF), TGF, Fibroblast growth factor (FGF), PDGF, HGF and ILs	[52]
Horse Peripheral blood MSC	Equine dermal fibroblasts	Increased migration. Promoted in vitro wound healing.	Not identified	[53]
Human BM-MSC	Peripheral blood mononuclear cells	Immunosuppressive. Decreased secretion of pro-inflammatory cytokines and increased secretion of anti-inflammatory cytokines. Increased ratio of Th2/Th1.	Not identified	[54]
Mouse BM-MSC	Excisional wounds (mice)	Increased macrophage polymerisation and re-epithelialisation. Improved wound healing.	Vascular endothelial growth factor (VEGF), IGF-1, Epidermal growth factor (EGF), KGF, Ang-1, SDF-1, Macrophage Inflammatory Protein (MIP-1α), erythropoietin	[36]
Human BM-MSC	Partial-thickness burn injury (rats)	Increased collagen deposition, cell proliferation and angiogenesis.	Not identified	[37]
Human BM-MSC	Full-thickness burn injury (rats)	Increased number of fibroblasts. Accelerated wound closure. Promoted angiogenesis and collagen deposition.	bFGF	[38]
Rat BM-MSC	Chronic wounds (rats)	Increased re-epithelialisation. Improved collagen deposition. Promoted wound closure.	Not identified	[39]
ADPSC	Full-thickness wounds (rats)	Accelerated wound closure along with faster re-epithelialisation.	VEGF, EGF	[55]
Human DPSC	Excisional wound splint model (mice)	Promoted proliferation and migration of fibroblasts. Accelerated collagen synthesis. Promoted healing.	Not identified	[56]
Human UC-MSC	Diabetic wounds (mice)	High blood vessel density. Improved healing. Higher levels of PDGF, VEGF and KGF expression in treated wounds.	Not identified	[57]
Human WJ-MSC	Excisional wounds (mice)	Increased cell proliferation and migration. Promoted wound healing.	Not identified	[58]
Human WJ-MSC	Radiation-induced cutaneous wounds (rats)	Accelerated healing.	Not identified	[59]
Human ADSC	Fractional carbon dioxide laser resurfacing (Human)	Reduced trans-epidermal water loss and accelerated healing.	TGFβ-1, VEGF, FGF, HGF, PDGF	[60]

**Table 2:** Soluble factors in MSC-S related to wound healing [36,40,61,62].

Growth Factors	Inflammatory Proteins	ECM Proteins	Angiogenic Factors
PDGF	IL-1	MMP-1	VEGF
IGF-1	IL-8	MMP-2	ANG-1
EGF	IL-10	MMP-3	ANG-2
FGF	IL-6	MMP-7	PDGF
Granulocyte-colony stimulating factor (G-CSF)	Tumour necrosis factor alpha (TNF)	TIMP-1	MCP-1
GM-CSF	Leukemia inhibitory factor (LIF)	TIMP-2	TGF-β1
HGF	IL-11	ICAM	FGF
PGE2	MCP-1	Elastin	EGF
TGF-βs	PGE2	Collagens	CXCL5
VEGF	IL-9	Decorin	MMPs
KGF	IL-13	Laminin	TGF-α

## Abbreviations

**ADSC**	Adipose-derived stem cells
**Ang**	Angiopoietin
**BM-MSC**	Bone marrow mesenchymal stem cell
**CD**	Cluster of differentiation
**DPSCs**	Dental pulp derived stem cells
**ECM**	Extracellular matrix
**EGF**	Epidermal growth factor
**FGF-2**	Fibroblast growth factor-2
**G-CSF**	Granulocyte - colony stimulating factor
**GM-CSF**	Granulocyte-macrophage colony-stimulating factor
**HGF**	Hepatocyte growth factor
**ICAM**	Intercellular adhesion molecule 1
**IFN-γ**	Interferon-gamma
**IGF**	Insulin-like growth factor
**IL**	Interleukin
**IL1-RA**	Interleukin-1 receptor antagonist
**KGF**	Keratinocyte growth factor
**LIF**	Leukemia inhibitory factor
**MCP-1**	Monocyte chemoattractant protein-1
**MHC**	Major histocompatibility complex
**MMP**	Matrix metalloproteinase
**MSCs**	Mesenchymal stem cells
**MSC-S**	Mesenchymal stem cells secretome
**PDGF**	Platelet derived growth factor
**PD-L1**	Programmed death-ligand 1
**SDF-1**	Stromal cell-derived factor-1
**TGF**	Transforming growth factor
**Th1**	Type 1 T helper cell
**Th2**	Type 2 T helper cell
**TIMP-1**	Tissue inhibitor of metalloproteinase
**TNF**	Tumour necrosis factor alpha
**UC-MSCs**	Umbilical cord-derived mesenchymal stem cells
**VCAM**	Vascular cell adhesion protein
**VEGF**	Vascular endothelial growth factor
